# Pediatric thyroid cancer: a retrospective analysis of 42 cases

**DOI:** 10.3389/fonc.2026.1783897

**Published:** 2026-07-01

**Authors:** Jian Chen, Yu-Ting Yin, Chao Gui

**Affiliations:** 1 Department of Head and Neck Surgery, Hubei Cancer Hospital, Tongji Medical College, Huazhong University of Science and Technology, Wuhan, Hubei, China; 2 Department of Gastrointestinal Surgery, Hubei Cancer Hospital, Tongji Medical College, Huazhong University of Science and Technology, Wuhan, Hubei, China

**Keywords:** extrathyroidal invasion, lung metastasis, lymph node metastasis, pediatric thyroid cancer, surgery

## Abstract

Pediatric thyroid cancer is rare. This study summarizes the clinical characteristics, treatment modalities, and therapeutic outcomes of pediatric and adolescent patients (≤18 years) with this malignancy treated at our institution. A retrospective analysis was conducted on 42 patients who underwent surgical treatment between 2018 and 2023. The cohort was predominantly female, comprising 34 girls and 8 boys. Postoperative histopathological analysis revealed 35 cases of papillary carcinoma, 4 of follicular carcinoma, and 1 each of mixed papillary-follicular carcinoma, medullary carcinoma, and poorly differentiated carcinoma. In 26 cases (61.9%), tumors exceeded 2 cm in diameter. The cervical lymph node metastasis rate was 76.2% (32/42), with 6 cases (14.3%) exhibiting lung metastasis. Surgical intervention was determined based on lesion characteristics and lymph node status. As of October 2025, patient follow-up ranged from 24 to 94 months, with a median duration of 61 months. Three patients (7.1%) underwent repeat neck dissection for recurrent cervical lymph node disease. No deaths occurred during the median follow-up period of 61 months. Statistical analysis suggested that extrathyroidal invasion is a significant factor influencing cervical lymph node metastasis in thyroid cancer, whereas bilateral involvement, multifocality, and extrathyroidal invasion are prominent indicators of lung metastasis. In summary, pediatric thyroid cancer predominantly affects females. Papillary carcinoma is the primary histological type, with tumors tending to be larger in diameter and exhibiting high rates of cervical lymph node and pulmonary metastasis. Nonetheless, with aggressive treatment, the overall prognosis remains favorable.

## 1 Introduction

Pediatric thyroid cancer (TC) is relatively uncommon, with an incidence rate significantly lower than that observed in adults. However, over the past 20 years, the diagnosis of pediatric TC has risen significantly in the United States, making it the most common pediatric endocrine malignancy, with an estimated annual incidence of 1.4 cases per 100,000 children (1, 2). Within this population, the frequency of TC increases with age and is tenfold higher in adolescents than in young children (3). Moreover, US National Cancer Database analysis indicates that children diagnosed before age 11 present with higher tumor stages and a greater risk of lymph node and distant metastasis (4). The most common initial symptom of pediatric TC is a painless neck mass. Consequently, the palpation of a firm nodule or enlarged lymph nodes during physical examination should prompt further investigation. For the initial diagnostic assessment of TC, ultrasound remains the preferred imaging modality.

TC in children and adolescents differs significantly from that in adults regarding clinical presentation, pathophysiology, treatment, and prognosis. While thyroid nodules are less common in children than in adults, their malignancy rate is significantly higher in the pediatric population, reaching up to 26.4% compared to approximately 5% in adults (5). In contrast to adult cases, pediatric TC is characterized by aggressive biological behavior at initial diagnosis, with patients often presenting with local invasion, lymph node metastasis, and distant metastasis. Despite this advanced initial presentation, long-term survival rates are higher in children. Additionally, although the incidence of pediatric TC has increased, the survival rate remains very high, approaching 100% (1).

Surgery is the primary therapeutic intervention for pediatric TC, with procedures ranging from lobectomy to total thyroidectomy (TT) and cervical lymph node dissection. The objective of this study was to summarize and analyze data on pediatric TC cases at our specialized TC center and evaluate treatment outcomes. We sought to characterize the features of pediatric TC and share treatment experiences, aiming to inform future clinical practice.

## 2 Subjects and methods

This retrospective cohort study was approved by the Institutional Review Board of Hubei Cancer Hospital. The study population consisted of 42 pediatric TC patients aged 18 years or younger who underwent surgical treatment at the Department of Head and Neck Surgery between 2018 and 2023.

In this investigation, active treatment was defined as any intervention specifically targeting the tumor with the goal of curing the disease or alleviating symptoms, including (i) surgical resection of the primary tumor or metastases, (ii) local treatment such as radiation therapy or ablation therapy, and (iii) systemic treatment involving targeted therapy, chemotherapy, or immunotherapy. Patients receiving only levothyroxine for thyroid-stimulating hormone suppression or best supportive care were classified as not receiving aggressive treatment. All pediatric patients were thoroughly evaluated *via* neck ultrasound and clinical examination. The clinical parameters assessed included patient age, gender, tumor size, focality, lateralization, extrathyroidal invasion, and distant lung metastasis.

Patients were excluded from the analysis if they met any of the following criteria: (i) did not undergo surgery, such as those who received only radioactive iodine (RAI) therapy, external beam radiation therapy (EBRT), systemic therapy, or best supportive care; (ii) received preoperative neoadjuvant therapy, which may alter the pathological assessment of extrathyroidal invasion; (iii) had incomplete clinical or pathological data; or (iv) were lost to follow-up.

### 2.1 Statistical analysis

Statistical analyses were performed using SPSS v.23 software (IBM, Armonk, NY, USA). Patients were divided into two groups based on the histopathological results for cervical lymph node metastasis (positive *vs*. negative). The cohort was further stratified according to the presence or absence of lung metastasis. Group differences were evaluated using Fisher’s exact test, with the threshold for statistical significance established at *P* < 0.05.

## 3 Results

### 3.1 Patient characteristics

The characteristics of the 42 patients included in this study are shown in **Table 1**. Most patients were female (34/42, 81%). The median age at the time of surgery was 15.7 years (range: 7–18), with 16 cases (38.1%) aged 15 years or younger and 26 cases (61.9%) older than 15 years. The predominant histological subtype was papillary thyroid carcinoma (PTC) (35 cases, 83.3%), followed by follicular thyroid carcinoma (FTC) (4 cases, 9.5%), and PTC combined with FTC, medullary thyroid carcinoma (MTC), or poorly differentiated thyroid carcinoma (PDTC) (one case each [2.4%]).

**Table 1:** Clinicopathological characteristics of the pediatric thyroid cancer patients.

Variable	Distribution (*n* = 42)	Percentage (%)
Age, mean (± SD)^a^	15.7 (± 2.7) range (7–18)	
Age		
≤15	16	38.1
15–18	26	61.9
Sex		
Female	8	19.0
Male	34	81.0
Hisopathologic subtype		
PTC	35	83.3
FTC	4	9.5
PTC+FTC	1	2.4
MTC	1	2.4
PDTC	1	2.4
Tumor size (cm)		
≤2	16	38.1
>2	26	61.9
Focality		
Unifocal	24	57.1
Multifocal	18	42.9
Side		
Unilateral	27	64.3
Bilateral	15	35.7
Extrathyroidal extension		
No	27	64.3
Yes	15	35.7
N staging		
N0	10	23.8
N1a	15	35.7
N1b	17	40.5
M staging		
M0	36	85.7
M1 (lung)	6	14.3

PTC, papillary thyroid carcinoma; FTC, follicular thyroid carcinoma; MTC, medullary thyroid carcinoma; PDTC, poorly differentiated thyroid carcinoma.

Lymph node metastasis was detected in 32 patients (76.2%), involving the central zone (CLN) in 31 cases and the lateral neck (LLN) in 17 cases. Lung metastasis was identified in 6 cases (14.3%). A total of 18 cases (42.9%) exhibited multifocal lesions. In 16 cases (38.1%), the maximum tumor diameter was ≤2 cm. Bilateral cancer foci and extrathyroidal extension (ETE) were each present in 15 cases (35.7%). Regarding surgical management, one patient (2.4%) underwent unilateral thyroid lobectomy, 12 (28.6%) received unilateral lobectomy with isthmusectomy and CLN dissection, 15 (35.7%) underwent TT with CLN dissection, and 14 (33.3%) underwent TT combined with both CLN and LLN dissection. Postoperative RAI therapy was administered in 16 cases (38.1%).

Rapid intraoperative tests failed to provide a definitive diagnosis of FTC in four patients with FTC and one with mixed FTC and PTC. Following postoperative routine histopathological confirmation, two patients with capsular or vascular invasion underwent reoperation to complete TT and lymph node dissection. One patient with a single-focus capsular tumor was managed with regular follow-up observation after unilateral lobectomy. In another case involving a 4.5 cm solitary tumor, management included unilateral lobectomy and isthmus resection with CLN dissection. No recurrence was observed during follow-up for these four cases. One patient with mixed FTC and PTC had a follicular tumor located on the left side and a confirmed right-sided PTC, and underwent TT with CLN dissection. However, the patient later developed LLN and lung metastasis, requiring LLN dissection and RAI therapy.

A 14-year-old female patient with MTC presented with a solitary right-sided lesion measuring 3.2 cm in diameter. Rapid intraoperative diagnosis indicated that malignancy was present, with MTC not excluded. Although the preoperative calcitonin level was 9.1 pg/mL (reference range: 0–6.4 pg/mL), preservation of the normal left thyroid lobe was strongly requested by the patient’s parents. Thus, the procedure was limited to right thyroid lobectomy and isthmusectomy combined with CLN dissection. Postoperative routine histopathology confirmed the diagnosis of MTC, with metastasis identified in one CLN. No recurrence has occurred during 81 months of follow-up.

Another 14-year-old female patient with PDTC presented with a tumor measuring 1.5 cm in maximum diameter and underwent TT combined with CLN and LLN dissection. The tumor demonstrated ETE, along with both vascular and lymphatic carcinomatous emboli. Postoperatively, the patient received chemotherapy and intensity-modulated radiotherapy (IMRT) to the neck, with no recurrence being observed at 80 months of follow-up.

Six patients presented with lung metastasis, including five with PTC and one with PTC combined with FTC. All five patients with PTC underwent RAI therapy. During follow-up, two patients exhibited slow progression of lung metastases, with one receiving oral targeted therapy. Two patients showed stable disease, while two demonstrated a reduction in the size of the lung lesions.

Three patients experienced recurrent cervical lymph node disease and underwent neck lymph node dissection to remove the cancer foci. Among these cases, one seven-year-old patient—the youngest in the cohort—experienced three recurrences in the neck lymph nodes and received three courses of RAI therapy. This case was eventually classified as iodine-refractory TC and was treated with oral targeted therapy (anlotinib). At 92 months of follow-up, the disease remained stable, with no evidence of further recurrence.

The median follow-up duration for the 42 patients was 61 months (range: 24–94), and no deaths were observed during this period.

### 3.2 Factors significantly associated with cervical lymph node metastasis

As shown in **Table 2**, the cervical lymph node metastasis rate was 100% in cases with ETE *versus* 63% in those without ETE. Fisher’s exact test revealed that this difference was statistically significant (*P* = 0.007), indicating that patients with ETE are more likely to develop cervical lymph node metastasis. In contrast, the cervical lymph node metastasis rate for patients aged 15 years or younger (75%) was comparable to that of patients older than 15 years (77%) (*P* = 1.000). Similarly, no significant difference in the cervical lymph node metastasis rate was observed between male and female patients (100% *vs*. 70.6%) (*P* = 0.165). The lymph node metastasis rate for multifocal TC (83.3%) was not significantly different from that for single-focal lesions (70.8%) (*P* = 0.473). The rate of lymph node metastasis for tumors ≤2 cm (81.3%) did not significantly differ from that for tumors >2 cm (73.1%) (*P* = 0.715). Similarly, the lymph node metastasis rate for bilateral cancer foci (86.7%) was not significantly different from that for unilateral foci (70.4%) (*P* = 0.286).

**Table 2:** Clinical characteristics of pediatric thyroid cancer patients with and without neck lymph node metastasis.

Variable	LN(−) (*n* = 8)	LN(+) (*n* = 24)	Fisher’s exact test *P*-value
Age			
≤15	4	12	1.000
15–18	6	20	
Sex			
Female	10	24	0.165
Male	0	8	
Tumor size (cm)			
≤2	3	13	0.715
>2	7	19	
Focality			
Unifocal	7	17	0.473
Multifocal	3	15	
Side			
Unilateral	8	19	0.286
Bilateral	2	13	
Extrathyroidal extension			
No	10	17	0.007
Yes	0	15	

LN, lymph node.

#### 3.2.1 Multivariable analysis

Multivariable logistic regression analysis showed that, after adjusting for age and tumor size, none of the studied variables—including ETE (*P* = 0.998), age (*P* = 0.720), and tumor size (*P* = 0.736)—were significantly associated with cervical lymph node metastasis (*P* > 0.05).

In the univariable analysis, ETE was associated with lymph node metastasis, which is consistent with previous reports. However, this association lost statistical significance after adjusting for age and tumor size. This outcome may be attributed to the relatively small sample size, which included only 15 patients with ETE, thereby resulting in insufficient statistical power. Although ETE was not identified as an independent predictor in this specific study cohort, it remains a clinically significant factor warranting further investigation in larger studies.

### 3.3 Factors significantly associated with lung metastasis in TC

**Table 3** details the results of Fisher’s exact test analysis for lung metastasis. The lung metastasis rate of 33.3% in patients with multifocal TC was significantly different from the 0% observed for unifocal TC (*P* = 0.007). Similarly, significant differences were identified for bilateral *versus* unilateral TC (40% *vs*. 0%) (*P* = 0.001), TC with *versus* TC without ETE (40% *vs*. 0%) (*P* = 0.001), and TC with *versus* TC without LLN metastasis (35.3% *vs*. 0%) (*P* = 0.002). These findings indicated that multifocality, bilateral involvement, ETE, and LLN metastasis are associated with a higher incidence of lung metastasis. In contrast, patient age (≤15 years: 12.5% *vs*. >15 years: 15.4%; *P* = 1.000), gender (males: 12.5% *vs*. females: 14.7%; *P* = 1.000), and tumor diameter (≤2 cm: 0% *vs*. >2 cm: 23.1%; *P* = 0.067) showed no significant correlation with pulmonary involvement.

**Table 3:** Clinical characteristics of pediatric thyroid cancer with and without lung metastasis.

Variable	LM(−) (*n* = 36)	LM(+) (*n* = 6)	Fisher’s exact test *P*-value
Age			
≤15	14	2	1.000
15–18	22	4	
Sex			
Female	7	1	1.000
Male	29	5	
Tumor size (cm)			
≤2	16	0	0.067
>2	20	6	
Focality			
Unifocal	24	0	0.004
Multifocal	12	6	
Bilaterality			
No	27	0	0.001
Yes	9	6	
Extrathyroidal extension			
No	27	0	0.001
Yes	9	6	
Lymph node			
−	10	0	0.308
+	26	6	
LNN			
N0, NIa	25	0	0.002
N1b	11	6	

LM, lung metastasis; LLN, lateral neck lymph node metastasis.

### 3.4 Risk stratification and recurrence analysis

Risk stratification for recurrence was performed according to the 2015 American Thyroid Association (ATA) guidelines, which are based on pathological data including tumor size, extrathyroidal invasion, lymph node status, the number and size of involved lymph nodes, and surgical margins. Of the 42 patients, 40 were included in this stratification analysis; one case of MTC and one case of PDTC were excluded. The remaining patients were divided into low-risk (*n* = 15), intermediate-risk (*n* = 13), and high-risk (*n* = 12) groups, with the detailed results of the stratification analysis presented in **Table 4**.

**Table 4:** Distribution of patients by ATA recurrence risk and outcomes.

ATA risk	*N* (%)	Lymph node recurrence	New lung metastases
Low	15 (37.5%)	0	0
Intermediate	13 (32.5%)	0	0
High	12 (30.0%)	3	3

ATA, American Thyroid Association.

A total of six cases of disease recurrence or progression were observed, comprising three cases of cervical lymph node recurrence and three cases of distant lung metastasis, all of which occurred exclusively within the high-risk group. Among the 25 patients classified into the intermediate- and high-risk groups, six (24%) experienced recurrence, whereas no recurrence was observed among the 15 patients in the low-risk group. Analysis using Fisher’s exact test revealed that the difference was statistically significant (*P* = 0.046), indicating that classification into the intermediate- and high-risk groups is associated with a higher recurrence rate.

## 4 Discussion

Pediatric TC is rare, accounting for only 0.5% to 3% of all childhood malignancies (6). The annual incidence rate of DTC among children is 0.54 cases per 100,000 population (3). Consistent with the 2015 ATA guidelines, in this study, we defined the age range for childhood and adolescent TC as 0 to 18 years (7). Data from the Surveillance, Epidemiology, and End Results database of the National Cancer Institute of the United States indicate that the overall incidence of TC among adolescents increased by approximately 1% annually from 2015 to 2019 (8). Furthermore, the incidence of different subtypes of TC varies across age groups, with a significant increase observed after age 14, accounting for 74% of all cases (9). Consistent with these data, among the 42 patients in our cohort, 26 (61.9%) were older than 15 years of age.

According to the World Health Organization’s pathological classification criteria, PTC is the most prevalent subtype of TC in children and adolescents, accounting for approximately 90% of cases. At 5% to 10%, FTC is relatively rare in this population. MTC is very rare, while anaplastic thyroid carcinoma is extremely rare (10). Our cohort comprised 35 cases of PTC (83.3%), 4 cases of FTC (9.5%), and 1 case each (2.4%) of PTC combined with FTC, MTC, and PDTC. Thus, the predominant histological type was PTC, with MTC and PDTC being extremely uncommon. No cases of ATC were observed.

Compared to its adult counterpart, pediatric TC is more aggressive and more likely to metastasize to lymph nodes and distant organs. The lymph node metastasis rate has been reported to range from 60% to 80%, while the lung metastasis rate approximates 10% (11). Among our patients, 32 (76.2%) exhibited lymph node metastasis, while 6 (14.3%) demonstrated distant (lung) metastasis. These findings are consistent with previously reported results.

Most pediatric patients present with a neck mass or enlarged cervical lymph nodes. Relative to those in adults, thyroid tumors in children tend to have a larger diameter at diagnosis. In our cohort, 26 cases (61.9%) involved tumors exceeding 2 cm in diameter, with most presenting as palpable thyroid masses or enlarged lymph nodes.

Current primary treatment modalities for pediatric TC include surgery, RAI therapy, hormone suppression therapy, and molecular targeted therapy. The main therapeutic goal in pediatric TC is to achieve a complete cure, prioritizing both overall survival and recurrence-free survival.

However, controversy persists regarding the optimal extent of surgical resection for pediatric and adolescent patients with low-risk PTC (12). Regarding the rationale for subtotal thyroidectomy, preserving part of the thyroid lobes in developing children both retains some thyroid function and reduces the risk of injury to the parathyroid glands and the recurrent laryngeal nerve. Furthermore, the endocrine functions of the thyroid gland are essential for the growth and development of children and adolescents. No study to date has confirmed that exogenously administered thyroxine can fully substitute for endogenous production, and it remains unclear whether TT affects the overall growth and development of the pediatric population. Consequently, some patients opt for a more conservative surgical approach to avoid potential complications and lifelong dependence on medication. Wang et al. demonstrated that the extent of resection is not an independent risk factor for recurrence and recommended partial resection to avoid potential complications following TT (13). Children with PTC have a high survival rate, with no significant differences in mortality reported among different surgical approaches (14). A meta-analysis of 10 studies involving 17,082 patients showed that the five-year recurrence-free survival rates for lobectomy and total resection were comparable in patients with low- to intermediate-risk PTC (15). For T3b DTC, the 2025 ATA guidelines recommend a TT; however, a study of 6,920 patients from the SEER cohort showed that a lobectomy is associated with equivalent survival outcomes to a TT, and the extent of surgery contributes very little to predicting prognosis, accounting for only 1.5% to 1.8% of the variance (16).

Compared with subtotal resection, total resection is associated with a higher rate of postoperative complications (17). The incidence of permanent recurrent laryngeal nerve injury is approximately 1% to 2% following TT and less than 1% following lobectomy (18). In a study of patients with locally advanced (cN1b) PTC, Li et al. found that the incidence of hypoparathyroidism was significantly lower in the lobectomy group than in the total thyroidectomy group (0% *vs*. 11.5%, *P* < 0.001). Furthermore, lobectomy achieved a significantly lower incidence of unsatisfactory TSH control than total thyroidectomy (5.9% *vs*. 20.8%, *P* = 0.006), while recurrence-free survival remained comparable between the two groups (19).

However, another perspective holds that because pediatric TC is highly aggressive and prone to recurrence, complete resection may be necessary to reduce recurrence rates and improve prognosis. A 40-year follow-up study of 215 pediatric TC patients revealed that the local recurrence rate following TT was 6%, which was significantly lower than the rate of 35% observed after unilateral lobectomy (*P* < 0.001) (20). It is therefore believed that total thyroidectomy reduces the risk of local recurrence compared to lobectomy. For pediatric patients undergoing surgical treatment for well-differentiated thyroid cancer, TT is recommended; performing a procedure less extensive than TT in a single operation significantly increases the need for reoperation, whereas TT does not result in a significant increase in surgical complications (21). If the surgery is performed by an experienced surgeon, the incidence of surgical complications can be further reduced (22). Conversely, among patients who undergo a completion TT following an initial near-TT, there is an increased risk of complications, particularly hypoparathyroidism and recurrent laryngeal nerve injury (23). Furthermore, after a TT, the iodinated TC lesions are the only sites in the body that absorb and accumulate radioactive iodine. RAI therapy acts as a form of internal radiation, killing microscopic cancerous lesions and metastases left behind during surgery, thereby reducing the rates of tumor recurrence and metastasis.

TT enhances the effectiveness of RAI therapy and helps monitor serum thyroglobulin levels more reliably, thereby improving patient follow-up (24). Among the 42 patients in our group, 29 (69%) underwent TT, with 3 (10.3%) experiencing neck lymph node recurrence. In contrast, the 13 patients (31%) who underwent lobectomy showed no cervical lymph node recurrence during follow-up. Analysis using Fisher’s exact test indicated that the recurrence rate did not significantly differ between the two groups (*P* = 0.540). Among patients who underwent TT, six (20.7%) developed lung metastasis, whereas no lung metastasis occurred in patients who underwent lobectomy. Again, Fisher’s exact test showed that the incidence of lung metastasis was comparable between the two groups (*P* = 0.153). These data suggest that the surgical approach did not significantly influence the rates of cervical lymph node recurrence and lung metastasis. This discrepancy relative to extant literature may stem from insufficient follow-up duration and sample size limitations. In summary, surgical approaches should be tailored to individual examination findings, while uniformly performing TT may represent an overly aggressive approach for pediatric patients (25).

FTC in children typically presents as minimally invasive (90% of cases), exhibiting lower aggressiveness than its adult counterpart. Additionally, minimally invasive FTC carries a favorable prognosis. For such patients, a lobectomy with isthmus resection is an appropriate treatment option (26). Diagnostic challenges persist because FTCs frequently exhibit ultrasonographic features similar to those of benign nodular goiters, and are often classified as Thyroid Imaging, Reporting, and Data System (TI-RADS) 3 or 4 (27). Additionally, preoperative fine-needle aspiration cytology (FNAC) and intraoperative frozen section analysis often fail to accurately establish the pathological nature of the lesion (28, 29). Given these challenges, achieving early pathological confirmation and diagnosis remains difficult. Consequently, many patients with larger tumors do not receive an initial diagnosis of FTC and, therefore, do not undergo TT during their initial surgery. Postoperative paraffin histopathology often confirms FTC, raising the question of whether a second surgery is required. In our cohort, rapid intraoperative frozen section testing failed to definitively diagnose FTC in four cases and FTC combined with PTC in one case. Diagnosis was eventually confirmed based on postoperative routine histopathology. Following these results, two patients exhibiting capsular or vascular invasion underwent reoperation, while two low-risk patients opted for periodic follow-up observation. None of these four cases showed recurrence during follow-up. The patient presenting with FTC combined with PTC underwent TT with CLN dissection during the initial surgery. During follow-up, left LLN metastasis and lung metastasis developed, requiring a left LLN dissection and RAI therapy; the patient’s condition is currently stable.

PDTC is a rare type of thyroid malignancy, with a reported incidence ranging from 2% to 15% among all TCs (30). Its morphological characteristics and clinical behavior are typically considered intermediate between DTC and ATC. Currently, surgery is the preferred therapeutic modality for PDTC. TT with complete macroscopic resection achieves satisfactory local control, with five-year rates reaching 81% in PDTC patients, whereas treatment failure in most cases is due to distant metastasis (31). For patients with PDTC who have T3 or T4 tumors or cervical lymph node involvement, EBRT, specifically IMRT, is recommended to address locally residual disease or a high risk of recurrence (30, 31). PDTC carries a poor prognosis, primarily due to advanced staging at diagnosis and frequent resistance to RAI therapy (32). Our cohort included a 14-year-old female with PDTC who underwent TT alongside CLN and LLN dissection. Pathological assessment revealed ETE, as well as vascular and lymphatic carcinomatous emboli. Metastasis was confirmed in 5 CLNs and 15 LLNs, signifying a high risk of recurrence. Postoperative chemotherapy and IMRT to the neck achieved good local control, and no recurrence was observed at 80 months of follow-up.

Prophylactic CLN dissection is essential for pediatric TC, with complication rates typically maintained at a very low level. Standardized and thorough CLN management is necessary to avoid reoperation in the central region. A 2022 study by Ngo et al. (33) found that the CLN metastasis rate in pediatric and adolescent patients with PTC reached 83.3%, with approximately 62.5% of patients also experiencing LLN metastasis. This high CLN metastasis rate justifies a more aggressive approach to CLN dissection in pediatric patients than in adults. In the present cohort, 32 cases (76.2%) presented with lymph node metastasis. Specifically, 31 cases (73.8%) exhibited CLN metastasis, 17 (40.5%) demonstrated LLN metastasis, and 16 (38.1%) displayed involvement of both regions. In our study, CLN dissection (Level VI) was performed as a prophylactic measure; however, in cases where imaging studies or FNAC clearly indicate the presence of lateral neck lymph node metastasis, LLN dissection is performed.

RAI therapy is primarily used for pediatric patients at moderate to high risk, particularly for inoperable residual local lesions, metastatic lymph nodes, and iodine-avid distant metastases. A systematic review by Pawelczak et al. (34) on pediatric and adolescent patients with DTC and lung metastases reported that postoperative RAI therapy and thyrotropin suppression resulted in a complete or partial response in 47.32% and 38.39% of cases, respectively, highlighting the high effectiveness of RAI therapy for lung metastases. Among the six patients with lung metastases in our group, five received RAI therapy. One patient experienced lung lesion progression, two showed stable disease, and two demonstrated a reduction in lung lesions. While these data indicate that RAI therapy is effective in treating lung metastases in DTC, longer-term follow-up is still required to monitor for delayed disease progression.

RAI-refractory TC in children and adolescents remains a significant clinical challenge and a crucial area for in-depth exploration. The advancement of molecular targeted therapies represents a shift toward personalized precision medicine for children and adolescents with TC, particularly those with advanced or metastatic disease. Such drugs hold promise for improving the prognosis of RAI-refractory disease by restoring RAI uptake capacity, while also reducing the tumor burden during adjuvant and neoadjuvant treatment phases (35). Targeted drugs, represented by tyrosine kinase inhibitors, offer distinct advantages over traditional chemotherapeutic agents, including higher specificity, improved therapeutic efficacy, fewer side effects, and convenience of administration.

In contrast to adult protocols, the management of advanced RAI-refractory TC in pediatric patients undergoing targeted therapy requires greater consideration of drug toxicity and potential teratogenic effects to ensure rational medication use. Currently, reference data regarding drug selection and dosage for pediatric targeted therapy remain insufficient. A recent clinical trial of selpercatinib involving pediatric patients with RET-altered solid tumors reported that among patients with measurable disease at baseline, those with PTC achieved an overall response rate of 100% (36). Furthermore, the U.S. Food and Drug Administration and the European Medicines Agency have approved sorafenib and lenvatinib for the treatment of locally recurrent or metastatic progressive iodine-refractory DTC. Lenvatinib demonstrates good tolerability and clinical activity in pediatric patients with advanced PTC and represents a viable therapeutic option for pediatric patients with PTC who are refractory to or ineligible for standard therapies (37).

Two patients in this cohort received postoperative targeted therapy. One of these patients was a seven-year-old girl who experienced three recurrences in her cervical lymph nodes despite undergoing a total thyroidectomy and lymph node dissection. Following three courses of postoperative RAI therapy, she was diagnosed with iodine-refractory TC. After obtaining informed consent, the patient began treatment with oral anlotinib at a dosage of 8 mg once daily, administered on a two-week-on, one-week-off schedule. Treatment began in 2018, and no significant adverse drug reactions were observed. Neck ultrasound examinations were performed every three or six months. After 92 months of follow-up, the patient’s condition remains stable, with no signs of recurrence.

Another case involved a 17-year-old female patient who developed lung metastases following TT for PTC, presenting with multiple nodules in both lungs, the largest of which measured approximately 0.8 cm in diameter. A follow-up examination three months after a single course of RAI therapy revealed disease progression, with the largest metastasis measuring approximately 1.1 cm in diameter. Once informed consent had been obtained, the patient began oral treatment with lenvatinib at a dosage of 20 mg once daily. Treatment began in 2023, and the pulmonary metastases gradually shrank and stabilized. Follow-up lung CT scans were performed every three to six months, and no significant drug-related adverse reactions were observed. At the 25-month follow-up, the largest diameter of the pulmonary metastases had decreased to approximately 0.4 cm.

### 4.1 Study limitations

The primary limitation of this study is the relatively small cohort size inherent to a retrospective, single-institution analysis. This limited sample size resulted in insufficient statistical power to perform robust multivariable analysis. Additionally, given the prolonged survival of pediatric patients treated with TT, extended follow-up durations are necessary to comprehensively investigate long-term recurrence rates and survival outcomes in these patients. Future research should prioritize larger multicenter sample sizes and extended follow-up periods.

## 5 Conclusions

Pediatric TC is a relatively uncommon malignancy that predominantly affects females. PTC is the primary histological subtype, characterized by larger tumor diameters and high rates of cervical lymph node and lung metastasis. Nevertheless, with aggressive treatment, the overall prognosis remains highly favorable. ETE is a significant factor associated with cervical lymph node metastasis in TC, while bilateral involvement, multifocality, ETE, and LLN metastasis are strong clinical indicators of lung metastasis. Finally, targeted therapy represents a highly viable therapeutic option for patients with RAI-refractory TC.

## Data Availability

The original contributions presented in the study are included in the article/supplementary material. Further inquiries can be directed to the corresponding author.
